# Income and wealth as determinants of voluntary private health insurance: empirical evidence in Spain, 2008–2014

**DOI:** 10.1186/s12889-020-09362-5

**Published:** 2020-08-19

**Authors:** Jaime Pinilla, Beatriz G. López-Valcárcel

**Affiliations:** 1 Department of Quantitative Methods for Economics and Management, University of Las Palmas de Gran Canaria, Campus de Tafira sn, 35017 Las Palmas de Gran Canaria, Spain; 2 Centre for Research in Health Economics, University Pompeu Fabra, Barcelona, Spain

**Keywords:** Voluntary private health insurance, Income and wealth semi-elasticities, Longitudinal data

## Abstract

**Background:**

Few studies have quantitatively estimated the income elasticity of demand of voluntary private health insurance (VPHI) in countries with a universal National Health Service. Most studies to date have uses cross-sectional data.

**Methods:**

In this paper we used a longitudinal database from the Bank of Spain to analyse the financial behaviour of approximately six thousand families per wave. We used three waves (2008, 2011 and 2014). We estimated income and wealth semi-elasticities of VPHI in Spain considering personal and family characteristics (age, sex, level of health, education, composition of the household), i.e. changes in the probability of buying VPHI as result of 1% change in income or wealth. We estimated cross-sectional models for each wave and longitudinal models for families remaining for at least two waves, taking account of possible selection bias due to attrition.

**Results:**

Cross-sectional models suggest that the income effect on the probability of buying a VPHI increased from 2008 to 2014. The positive impact was observed for, wealth. In 2008 a 1% increase in income is associated with an increase in the probability of having VPHI of 0.064 [95%-CI: 0.023; 0.104] - on the probability scale (0.1) – whereas in 2014, this effect is of 0.116 [95%-CI, 0.094; 0.139]. In 2011 and 2014 the wealth effect is not significant at 5%. The estimation of the longitudinal model leads to different results where both, income and wealth are associated with non- significant results.

**Conclusion:**

Our three main conclusions are: 1) Cross-sectional estimates of semi-elasticities of VPHI might be biased upwards; 2) Wealth is alongside income are economic determinants, of the decision to buy VPHI in high-income countries; 3) The effects of income and wealth on the probability of buying VHPI are neither linear nor log-linear. There are no significant differences among 60% of the most disadvantaged families, while the families of the two upper wealth quintiles show clearly differentiated behaviour with a higher probability of insurance.

## Background

In high income countries, health care systems are commonly categorized according to three systems: a national health service (NHS) system, a social insurance system, and a private insurance system. Countries like the United Kingdom and Spain are the most common examples of NHS systems, Germany has a social insurance system, and the United States (US) is an example of a country with a private insurance system [1].

Even in countries with a universal NHS, a number of individuals and families purchase private health insurance. According to the definition of the OECD Health Accounts System [2], “private health insurance comprises insurance schemes financed through private health premiums, i.e., payments that a policyholder agrees to make for coverage under a given insurance policy, where an insurance policy generally consists of a contract that is issued by an insurer to a covered person. Take up of private health insurance is often, but not always, voluntary (it may also be compulsory for employees as part of their working conditions).” Voluntary private health insurance (VPHI) is very prevalent in Europe [3], with a large variety of reasons for affiliating. Although VPHI has had, for many years, an important role in the health sector in low- and middle-income countries, it has traditionally been of minor importance in high-income countries with a universal public NHS and a welfare state. US is high income country with high importance of health insurances, but it has no universal public NHS.

The trend towards an increasing prevalence of VPHI is noticeable even in the Nordic countries, which tend to be benchmark countries in terms of their NHS and welfare states. Complementary VPHI plays a significant role in Denmark and in Finland, while supplementary VPHI is prominent in Norway and Sweden [4]. Although double insurance was initiated to respond to difficulties in access to the public system, once the private insurance market became established, it took on a life of its own, and as cultural patterns changed, the demand for private insurance remained strong. Another argument to explain the increasing trend towards VPHI relies on the progressive inequality in income distribution, particularly among the top earners [5]. As the level of economic inequality increases, it becomes more and more difficult for publicly provided insurance to satisfy the median citizen, as the targeted population is more and more heterogeneous. This may be the case in some OECD countries. Income becomes a paramount focus of interest when analysing the phenomenon of VPHI in developed countries.

The demand for VPHI depends on risk aversion, quality of and access to public services in the area. It also depends on the supply of private insurance in the region, its cost, and the health status of the population. Adverse selection and moral hazard, which may vary widely among individuals, influence in opposite directions the relationship between risks to health and the proclivity to by VPHI [6]. Individuals with worse health status have a higher probability of purchasing VPHI as they expect to use more services. But the price of the premiums increases with the probability of using healthcare services, thus reducing the probability of purchasing VPHI.

There are three types of VPHI: substitutive; supplementary; and alternative. In Spain, which has an almost universal NHS covering 99.1% of the population [7], there is a combination of supplementary/alternative VPHIs with a relatively low premium. In 2018 the average price of an annual premium was €760 [8] and the coverage tends to be limited to a range of specialized services associated with prevalent conditions. VPHI has therefore a doble role, providing an additional coverage for services already covered by NHS and also providing coverage for benefits not covered otherwise by the NHS as dental care in adults. VPHI is usually bought to avoid waiting times in the public NHS. According to the 2018 Healthcare Barometer [9], 78% of the individuals with double coverage declared that their main reason for buying VPHI was the waiting time. According to the Healthcare Barometer of Catalonia - one of the richest (it ranks the third in per capita income in 2018 [10]), and most populated regions in Spain – [11], 54% of households with income above €3000 per month had a VPHI coverage in 2017. In the highest-income district of the city of Barcelona, Sarriá-Sant Gervasi, 72% of the population had VPHI [12]. A study for Catalonia that used a pseudo-structural model to estimates the probability of buying VPHI conclude that there is a quality gap, and that the difference in the perceived quality between the private and public services (including waiting times) largely determines the likelihood of getting private insurance [13]. In the United Kingdom there is evidence that the probability of buying VPHI is also positively associated with waiting times in the public NHS, and private supply in a specific region is positively correlated with the median of the region and the year-specific public-sector waiting times [14]. If public insurance reduces the quality of healthcare available (longer access times) and healthcare is a normal good, it is expected that richer individuals will be willing to pay for private insurance.

In Spain, an increasingly active private health insurance sector is putting pressure on the health system [15]. There is an influential lobby that promotes the healthcare model offered to approximately two million civil servants in Spain, who are able to either choose between public (NHS) or private health insurance provided by a private profit-making company under an agreement with the civil servants mutual fund.

There is abundant literature about price and income elasticities of health expenditure, and about the price elasticities of private insurance, but references to income elasticities of private health insurance are scarce. Studies looking at the association between wealth and VPHI are scarce too, and most of them have focused on low- or medium-income countries and used ownership of a dwelling as an approximation of total real assets [16].

Few studies have quantitatively estimated the income and wealth semi-elasticities of the probability of purchasing private insurance, i.e., changes in the probability of buying VPHI derived from a 1% change in the continuous explanatory variable, and most studies on this topic have used cross-sectional data. In this paper we take advantage of a longitudinal database prepared by the Bank of Spain to analyse the financial behaviour of families. A fundamental characteristic of its sample is the over-representation of high-wealth households, while surveys of health and living conditions, being of proportional allocation, include very few well-off families. Another characteristic of this survey is that the question about private health insurance relates only to a policy voluntarily taken out by the family, excluding both the civil servants (approximately 1.8 million insurance coverages, 20% of the total, and insurance policies taken out) and insurance coverages paid by the employer (approximately 3.1 million, 34% of the total insurance coverages in 2017).

Furthermore, our data include a panel of three periods, 2008, 2011, 2014, from the beginning of the economic crisis until its end. Spain is a particularly interesting country because of the breadth and depth of the economic crisis. In order to provide a broader context, we have also worked with the previous waves (2002 and 2005) for the descriptive overview. The models with microdata focus on the 3 years for which there is longitudinal information.

We estimate income and wealth semi-elasticities of private insurance in Spain, taking account of personal and family characteristics (age, sex, level of health, education, composition of the household). We estimate cross-sectional models for each wave of the survey and longitudinal models for families that remain for at least 2 years in the panel, taking account of possible selection bias due to attrition. We consider income and absolute and relative wealth in the population distribution in each year (quintiles).

The next two sections of the paper describe the data and methods used in the analyses, followed by the results and discussion section. The article ends with a brief conclusion.

## Methods

Descriptive statistics (mean, median) of net income and wealth of Spanish households from 2002 to 2014 and 95% confidence intervals have been estimated. For each wave, we calculate crosstabs and bivariate tests (Chi-squared) between insurance coverage and relative income and wealth, defined by quintiles of equivalent income/wealth. Equivalent income/wealth is calculated using the modified OECD scale [18]. For those households with zero income and for those households with negative or zero wealth the corresponding logarithm was set equal to zero. Households are weighted according to their sampling weights.

Cross-sectional logit models for the 2008, 2011 and 2014 waves (*t* = 1.2 and 3 respectively) are used to estimate the probability of buying VPHI and to calculate cross-sectional income and wealth semi-elasticities and their 95% confidence intervals:

1 $$ P\left({Y}_{it}=1|{X}_{it}\right)=\mathrm{F}\left({\beta}_0+{\beta}_1\log \left({income}_{it}\right)+{\beta}_2\log \left({wealth}_{it}\right)+{\boldsymbol{\beta}}_3{\boldsymbol{Z}}_{it}\right) $$

where *Y* is the binary variable for having VPHI, and *X* includes log of income, log of wealth and a set of control variables (Z) measured for household *i* in year *t*: age, sex and educational level of the head of family (the head of the family is the person in the household who chiefly deals with the financial issues), percentage of members of the household with poor or very poor health, number of people in the household younger than 14 years of age, and expected future income. Lambda F(.) is the logistic cumulative probability function (cpf). The models use the sample weights defined by the sampling method.

These models check the observable variables in the survey (***Z***) that are likely to influence private insuring. The causation is through different mechanisms. For instance, premium influences negatively the probability of buying VPHI, ceteris paribus, but premiums are expected to vary with age, health status and size of the household.

Some relevant determinants of the decision about buying insurance, such as the place of residence (associated with availability of VPHI, prices and penetration of private insurance), are excluded from the survey, and there may also be unobserved characteristics of the household correlated with random error, such as risk aversion. So cross-sectional models applied independently to the three waves provide biased estimates. A priori, the sign of the bias is unclear. Omitting risk aversion from the equation would probably lead to a downward bias if risk aversion is positively related to the ownership of VPHI and negatively related to average income and wealth. If risk aversion is independent from income and wealth, the bias is likely to be positive. It is plausible that these omitted variables are positively correlated with income and wealth and that the bias of the cross-sectional estimates is therefore positive, so the models overestimate the effects of income and wealth on the probability of buying VPHI.

From the point of view of risk aversion, the uncertainty about the future plays an important role in the perceived need for health insurance. In our model we considered a variable related with the optimism for the future. This variable was derived from the question “Do you think that in the future your income will be higher, lower or the same as at present?”. We defined a dummy variable where “expected future higher income” is equal to 1 or 0 otherwise.

Optimism is the extent to which people hold favourable expectations for their future and it is expected to be correlated with more persistence in educational effort and higher later income. According to Caver [19], optimism is associated with taking proactive steps to protect health.

In non-linear regression models for cross-sectional data, bias from unobserved heterogeneity is particularly important if the researcher does not observe all the relevant independent variables that affect the outcome [20]. The unobserved heterogeneity can be dealt with in a number of ways. Because a subset of households is followed over time in our study, we propose to deal with the unobserved heterogeneity by estimating conditional fixed effect logit models. We estimated conditional fixed effects logit models for the longitudinal sample, checking for the same variables as in the cross-sectional models (1) and additionally including the inverse Mills ratio to account for the possibility of a selection bias associated with attrition from the panel.

Only 1524 of the 18,423 households in the cross-sectional database were surveyed in three waves, and 5247 households remained in the panel for at least two of the 3 years. In order to explore the possibility that the loss of individuals might be due to attrition, we estimated for each wave a probit selection equation for the probability of belonging to the longitudinal sample, with a set of explanatory variables that might cause attrition. We include in the list all the explanatory variables in eqs. (1):

2 $$ P\left({panel}_{it}|{X}_{it}\right)=\Phi \left({X}_t{\gamma}_t\right) $$

where Φ is the cpf of a standard normal and *γ*_*t*_ is a vector of coefficients for year t (t = 1,2,3).

From (2) we predict the probability of each household remaining in the study in each year, and we calculate the inverse Mills ratio (IMR), i.e. the standard normal density function evaluated in the estimated score (*X*_*it*_*γ*_*t*_) of the household divided by the estimated probability of remaining in the study. We use the new variable IMR as an additional regressor in the longitudinal equation, that is, a logistic model with the same *X* variables as in (1). The IMR derived from a first step probit selection equation has been used to correct for attrition bias. This procedure has been extensively used in panel models (see for instance Leigh, Ward and Fries [21] and Wooldridge [22]). In the original model proposed by Heckman [23] and in most applications, the second stage equation is linear, and it is estimated consistently by ordinary least squares, or alternatively both equations are jointly estimated by maximum likelihood, particularly when the second stage equation is nonlinear.

We estimate (2) with the sample of the households that were observed for at least two of the 3 years. The model includes fixed effects (*α*_*i*_) of the household to cancel out the unobserved individual heterogeneity that might bias the cross-sectional estimates:

3 $$ P\left({Y}_{it}=1|{X}_{it},{\alpha}_i,{IMR}_{it}\ \right)=\mathrm{F}\left({\beta}_0+{\beta}_1\log \left({income}_{it}\right)+{\beta}_2\log \left({wealth}_{it}+{\boldsymbol{\beta}}_3{\boldsymbol{Z}}_{it}+{\beta}_4{IMR}_{it}+{\alpha}_i\right)\right) $$

In order to estimate the longitudinal model, the households that did not change their insurance status while participating in the study do not provide information to the likelihood function, as in this case there is no variability within a subject, and therefore there is nothing to examine. They are automatically excluded from the estimation sample. The final sample is comprised of the 809 households (1928 observations) that either bought or cancelled health insurance at least once during the observation period.

In both cross-sectional and longitudinal logistic models, we estimate the marginal effects of income and wealth on the probability of buying VPHI as the Average of Partial Effects (APE) or semi-elasticities. The expression for income in the cross-sectional models is:

4 $$ \frac{\mathrm{\partial P}\left({Y}_{it}|{X}_{it}\right)}{\partial \log \left({\mathrm{income}}_{\mathrm{it}}\right)}={\beta}_1f\left({\beta}_0+{\beta}_1\log \left({income}_{it}\right)+{\beta}_2\log \left({wealth}_{it}\right)+{\boldsymbol{\beta}}_3{\boldsymbol{Z}}_{it}\right) $$

where *f*(.) is the density function of the logistic evaluated as the family’s estimated score. The expression (4) is evaluated for each family and averaged over families. It is not possible to estimate the semi-elasticities in the conditional fixed effect logit models for longitudinal data in the usual way as they depend on the fixed- effects, which in turn vary between individuals. We applied the transformation and method proposed by Kitazawa [24] to estimate the average semi-elasticities of *P*(*Y*_*iy*_ = 1| *X*_*it*_, *α*_*i*_) with respect to the regressors, and the corresponding standard errors and t-statistics. We used the Stata module developed by Silva [25].

Models (1)–(3) are estimated using relative income and wealth (quintiles) instead of the corresponding logarithms. For these models we compute odd-ratios for each level of income and wealth with reference to the first quintile (4). Estimates were calculated using the software package Stata 15.1 [26].

In order to check if the estimation of our model without prices of VPHI yields inconsistent and biased estimates of income and wealth semi-elasticities, we estimated an alternative model with imputed VPHI prices. To do this, we used data of the annual expenditure in private health insurance from the Household Budget Survey (EPF in Spanish), where a VPHI price was attributed to each family in our sample based on its composition, age and gender of the person who contributes most to the household income. Health insurance is defined in the EPF according to code 12.4.3.1 in the classification of individual consumption according to purpose (COICOP): “charges for private sickness insurance”. To avoid perfect multicollinearity, family composition and age and gender variables have been omitted in this model. Variations in VPHI prices due to geographical location, health plan choice and previous chronic conditions have not been considered.

### Data

We use microdata from the Survey of Household Finances (EFF in Spanish), conducted by the Bank of Spain with detailed longitudinal information about households, including income, wealth, debt and expenditure as well as a rich set of socioeconomic variables, attitudes to risk, and insurance behaviour. The income variable is calculated as the sum of labour and non-labour incomes for all household members. The wealth variable is calculated as the total household assets (real and financial assets) excluding public and occupational pension wealth minus total outstanding household’s liabilities. For more details on the definitions of the variables, see methodological notes of the survey [17]. Self-assessed health is recorded for each member of the household. There are five waves (2002, 2005, 2008, 2011 and 2014), including about 6200 households per wave. A fundamental characteristic of its sample is the over-representation of high-wealth households. A subset of households is followed over time. We use longitudinal information for 2008, 2011 and 2014 in those cases where data from specific household appears at least twice between 2008 and 2014. We also use cross-sectional information about all the individuals in each wave of the survey.

By analysing Spanish households longitudinally, we can use the variations in income and wealth during the economic crisis to estimate the effects of income and wealth on the probability to buy a VPHI. In the EFF, VPHI is defined as “a type of insurance whereby a private organization (the insurer) pays the medical costs of the insured if the insured becomes sick due to covered causes, or accidents. Private health insurances may be complements or substitutes of public health insurance”. The cross-sectional sample has 18,423 households (47,238 individuals), of which 6197 households (15,850 individuals) relate to 2008, 6106 households (15,852 individuals) to 2011 and 6120 households (15,536 individuals) to 2014. The longitudinal sample is comprised of the 5247 households that remained in the sample for at least 2 years between 2008 and 2014. Of these, 809 changed their insurance status during the period of study.

## Results

**Fig. 1:**
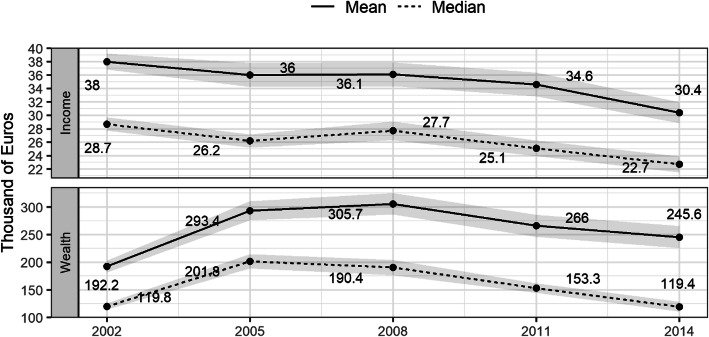
Median income and wealth Spanish households, 2002–2014. Source: Own elaboration using data from the Survey of Household Finances

From 2008, median income and median wealth of Spanish households decreased sharply (Fig. 1). The decrease in income was due to unemployment (the unemployment rate increased from 7.93% in the second quarter of 2007 to 24.47% in the second quarter of 2014), the general reduction of wages in the public sector in 2011 and the internal deflation through wages in the private sector. Net wealth decreased even more abruptly than income because of the property crisis after the bursting of the property bubble. The assets of many families lost value quickly during the years of economic crisis.

Table 1 shows descriptive univariate statistics for the cross-sectional samples. The figures in the table have been calculated using the sampling weights corresponding to each family and year. The distributions are similar for the three waves. Unweighted descriptive analyses (not reported) show older families (average age is approximately 60), with fewer women as heads of family and fewer children. As expected, due to the overrepresentation of richer families, net income and wealth are, on average, quite a lot higher in the unweighted sample than in the weighted sample. The same applies for the frequency of VPHI.

**Table 1:** Descriptive statistics cross-sectional samples

Variable	Categories	2008	2011	2014
Sex of the head of family (Female)		49.6%	45.1%	46.4%
Age of the head of family		52.9 (52.2–53.6)	53.9 (53.2;54.7)	54.5 (53.8–55.2)
Percentage of families whose head of family declares bad or very bad health in last 12 months		7.6%	8.7%	8.2%
Number of children (< 14 years) in the family	No children	73.3%	73.1%	73.4%
	1 child	15.2%	16.0%	16.0%
	2 children	10.0%	9.2%	9.0%
	> 2 children	1.5%	1.7%	1.6%
Educational level of the head of family	No studies or primary unfinished	38.8%	39.6%	37.3%
	Primary	17.0%	13.2%	13.6%
	Secondary	26.5%	27.5%	26.3%
	University	17.7%	19.7%	22.8%
Expected future income higher than current income		22.5%	20.2%	21.0%
Equivalent Income (in euros)		18,582 (17,846;19,317)	19,766 (18,923;20,609)	18,398 (17,550;19,246)
Equivalent wealth (in euros)		170,012 (159,715;180,308)	161,904 (151,921;171,886)	158,903 (143,670;174,136)
Estimated voluntary private health insurance prices (annual average expenditure per household in euros)		897.33 (881.96; 912.71)	849.48 (833.90; 865.06)	960.84 (942.97; 978.70)
Health Insurance	The family has health insurance	13.2%	14.5%	14.3%

For continuous variables, the table reports mean and linearized standard error. For categorical variables, % in each category. Calculations made with sampling weights

Table 2 shows the insurance status by quintiles of income and wealth for the whole sample each year (full cross-sectional samples). The association between insurance and both relative income and relative wealth is clear from this table. For instance, only 3.4% of the households in the poorest income quintile have VPHI in 2014, while 49.6% of the richest quintile do have insurance. In Table 2 we can also observe that the largest difference is from the third to the fourth quintile. The two most affluent quintiles, in income and in wealth, show far more proclivity to buy VPHI in Spain.

**Table 2:** Percentage of households with private insurance by income quintiles and wealth quintiles in the three waves

	Income			Wealth		
	2008	2011	2014	2008	2011	2014
Q1	3.4	3.8	3.4	6.9	10.0	8.7
Q2	6.9	9.2	7.1	9.6	10.0	9.8
Q3	10.8	11.9	12.8	14.6	13.8	13.1
Q4	25.4	29.6	27.7	22.6	29.6	30.3
Q5	35.8	38.9	49.6	44.4	48.3	46.8
Total	13.2	14.5	14.3	13.2	14.5	14.3

Calculations use cross-section sampling weights of households derived from the sampling design. Income and wealth are corrected by family size and composition with the OECD scale of equivalent income

Income and wealth semi-elasticities for each wave and for the panel (longitudinal model) are shown in Table 3, together with their respective 95% confidence intervals. Cross-sectional models suggest that the income effect on the probability of buying VPHI increased from 2008 to 2014, while the impact of wealth decreased. The effect of income is larger than that of wealth. In 2008 a 1% increase in income is associated with an increase in the probability of having VPHI of 0.064 - on the probability scale (0.1) - while in 2014 that effect is of 0.116. In 2008 an increase of 1% in wealth was associated with an increase of 0.015 in the probability of buying VPHI. In 2014, the effect wealth is not significant at a 5% level. The estimation of the longitudinal model leads to different results. Income and wealth are not significant. The estimated income semi-elasticity (0.076) is similar to the cross-sectional estimates. An increase in income of 1% is associated with an increase of 0.076 in the probability of buying VPHI. Wealth elasticity in the panel model (0.048) is higher than the cross-sectional estimates, and as in the cross-sectional models, the effect of wealth is smaller than the effect of income. The Mills ratio is not significant, suggesting that there is no attrition bias. The other covariates included in the model (table available on request) are not significant, with the exception of the percentage of members of the family declaring poor or very poor health (positive sign, significant at 10% but with a small coefficient), the education level of the head of the family, and the expectation of having a higher income in the future. The large variances obtained in the panel model are a consequence of the small sample and lack of within-family variations in most of the covariates such as sex, educational level, and family composition.

**Table 3:** Cross-section and panel logit modes. Estimation of income and wealth semi-elasticities of VPHI

Wave	N° of observations	Income	95% CI	Wealth	95% CI
2008	6197	0.064^a^	(0.023;0.104)	0.015^a^	(0.006;0.025)
2011	6084	0.080^a^	(0.050;0.109)	0.008^b^	(0.001;0.014)
2014	6116	0.116^a^	(0.094;0.139)	0.003	(−0.002; 0.009)
Panel	1928	0.076^c^	(−0.014; 0.168)	0.048^c^	(−0.009; 0.104)

The values in the table report the absolute change in the probability of having voluntary private health insurance if the income or wealth increases by 1%. All the models adjust by age, sex and education level of the head of the family, expected future income, number of children under 14 years of age in the household, and proportion of people in the household with bad or very bad health. The income and wealth of the household are in logarithms and adjusted by family composition according to the OECD scale. For the conditional fixed effects logit model (panel data) the estimates are average (semi) elasticities of *P*(*Y*_*iy*_ = 1| *X*_*it*_, *α*_*i*_), calculated following Kitazawa [22]. The last row contains the estimators of the panel model with household fixed effect

^a^significant at 1%

^b^significant at 5%

^c^significant at 10%

To account for this potential bias, we estimated our models using an imputed VPHI price for each family. The income and wealth semi-elasticities results have not changed significantly. Cross-sectional models suggest that the price effect (a 1% increase in price) is associated with a decrease in the probability of having VPHI of − 0.035 in 2008, while in 2014 that effect is of − 0.032, in 2011 the price effect is not significant. The impact of the other covariates is similar in all models. The Additional File 1 contain the estimates of this model. The estimation of price semi-elasticity in the longitudinal leads to non-significant results.

It may be that income and wealth do not have (log) linear effects on the probability, i.e. the relevant explanatory variable could be the relative rather than the absolute levels of wealth. In Table 4 we show the results of the models that include as independent variables the dummies for the quintiles of income and wealth (excluding the first quintile as reference) for the cross-sectional samples and for the panel model. The effect of income increases over time (according to the cross-sectional models) and it is much more intense for the two top quintiles Q4 and Q5 than for the bottom quantiles. The same result is derived from the panel model, although the odd-ratios are somewhat smaller than in the cross-sectional models. The Odd-Ratio (OR) for the two top income quintiles is 1.9 and 1.7, respectively. For the families in the fourth quintile of income, the relative probability of buying VPHI is almost double that of the poorest families.

**Table 4:** Cross-section and panel logit models. Estimates of Odd-Ratios (OR) of VPHI for relative income and wealth (by quintiles)

	Income				Wealth			
Wave	Q2	Q3	Q4	Q5	Q2	Q3	Q4	Q5
2008	1.6^c^	2.1^a^	4.5^a^	4.9^a^	1.3	1.8^a^	2.4^a^	5.3^a^
2011	1.9^a^	2.0^a^	4.3^a^	4.0^a^	0.9	1.1	2.9^a^	5.4^a^
2014	2.1^a^	2.6^a^	4.9^a^	8.9^a^	0.9	1.1	2.0^a^	3.2^a^
Panel	1.0	1.1	1.9^a^	1.7^b^	1.0	1.2	1.9^b^	2.6^a^

The values in the table are Odd-Ratios estimated with reference to the first quintile. All the models adjust by age, sex and educational level of the head of the family, expected future income, number of children under 14 years of age in the household, and proportion of people in the household with bad or very bad health. The income and wealth quintiles of the household have been calculated for each year

^a^significant at 1%

^b^significant at 5%

^c^significant at 10%

A similar effect is observed for wealth. According to the panel model, the ORs for the two wealthiest families are 1.9 and 2.6, respectively. The families in the top 20% by wealth have a probability of getting insurance that is two-and-a-half times greater than that of the families in the bottom 20%. As with income, the gap relates to the two top quintiles, while the three quintiles at the bottom of the wealth distribution do not differ significantly.

## Discussion

The study covers the period of the economic crisis, which imposed major changes in domestic economies in Spain. The richness of the longitudinal data has allowed the possibility of analysing the behaviour of families by comparing cross sectional and longitudinal models.

The three main contributions of this study are:

1. We have shown that cross-sectional estimates of income and wealth semi-elasticities of VPHI may be biased upwards (although panel estimates may have an upward bias too due to the small *T* problem [27]. As the large majority of studies published in the literature on this topic are based on cross-sectional data, our results provide a new contribution to the literature
2. Wealth is an economic determinant, together with income, for analysing the decisions to buy VPHI in high-income countries. However, studies of elasticities of wealth are very scarce. Wealth has been neglected in studies of health insurance for high-income countries, although it is usually considered in empirical studies for low- and middle-income countries. In studies in low and middle-income, wealth, absolute or relative, is usually introduced as a substitute of the economic status because it is easier to measure than income [28–31]. Some previous studies in the US and Europe have drawn attention to the importance of wealth when analysing the use of health services, regarding it as an even more sensitive indicator than income for older adults [32]. In the US it has been shown that assets, rather than income, are an important determinant of effective affordability of medical insurance [33]. However, to the best of our knowledge, ours is the first empirical study that quantifies the semi-elasticity of VPHI with respect to wealth using panel data. Wealth is closely correlated with income, but they are not measured in the same way. A study for 13 European countries estimates that each additional percentile in income distribution is associated with about 0.4 net wealth percentiles [34].
3. The effects of income and wealth on the probability of buying VHPI are neither linear nor log-linear. The position in the distribution is more important than the absolute level to explain the behaviour of families when buying insurance. There are no significant differences between the 60% of the less well-off families, while the families of the two upper quintiles show clearly differentiated behaviour, with a higher probability of VPHI.

One possible reason for the estimates in the categorical models (Table 4) to be much lower than when exploiting longitudinal information of the data may be measurement error in income and wealth quantiles. The bias due to measurement error in explanatory variables tends to be exacerbated in fixed effects model.

Our estimates of income semi-elasticities are larger than those calculated in studies in the United Kingdom, which were based on data for 10,729 individuals corresponding to five cross-sectional surveys between 1986 and 1991 [35]. These authors estimate the marginal effect of income at 0.0037, much lower than our estimates. In their model they do not adjust income by household composition, so it is possible that other explanatory variables of the household are also partially capturing the effect of income. The income semi-elasticities estimated by us are not comparable with previous studies in Spain which also estimate elasticities. The study of Costa and García that looks at data from 1999 [13], with a cross-sectional sample, estimates high income elasticities of approximately 1.22 for the total set of families in Catalonia, and even more for those that perceive low quality in the public sector. When using relative income and wealth data, results suggest non-linear effects, unlike a study conducted in Taiwan in which the effect of successive quintiles was monotonous [16]. Numerically, the ORs that were estimated in the Taiwan study for the most well-off quintile (OR = 2.5) are similar to ours.

As for the other explanatory variables, it should be noted that unlike in other studies, we did not find any significant relationship between the age of the head of the family and the probability of buying VPHI. In the panel models this could be due to little intrafamilial variability in the age of the head of family between waves.

This study contributes to the current debates about tax exemptions for private insurance and about the possible change of the public insurance model. VPHI has been considered equitable as the double-insured rich opt out to the private sector, leaving resources available to the poor, and at the same time the richer population contributes to subsidising public services through income taxes [36]. In some countries, employer-paid health insurance is subsidised under that opportunity cost argument. Several studies show that double cover causes an increase in private utilization that may overcompensate for the utilization of the public providers. In Italy the wealthier population replaces public consumption with private (opt out) [37], but in other countries it may happen that the double insured just consume private services without reducing the use of the public ones (top up) or at least consume more than comparable citizens with only the public cover. This seems to be the case for the elderly in Italy, Spain, Denmark, and Austria [38]. However, another study in Spain and based on the data from the National Health Survey suggests that people with VPHI “use the public health system less than individuals without double health insurance coverage” [39]. Our study shows that in Spain, the richest population (the fourth and especially the fifth income quintile) have a very high prevalence of VPHI. A tax exemption for health spending under the opportunity cost argument would have a regressive effect on the distribution of income.

This study also has some limitations. Although the conditional fixed effects control for household heterogeneity, there is still a risk of omitted variables that may cause bias, specially coming from supply side characteristics. Changes in the public health insurance coverage and quality of public health services (waiting times, access) are likely to be strong determinants of the decision to by private insurance. This fact makes it not possible to establishing a causal link between income, wealth, and insurance choice. Conditional fixed effects logit model for panel data faces the small *T* upward bias [26]. Although this might be a limitation, for studies looking only at handful time periods like ours, this method is usually preferred over the unconditional estimate as the bias tends to be smaller. We have no data on the geographical location of the people surveyed, the offer and on the waiting times of the public healthcare network in their areas of residence. These are important explanatory factor for buying VPHI [13, 14]. The sample size for the panel data logistic models is rather small because a considerable number of households did not change insurance status and were consequently removed from the estimated sample. Although our panel estimators are imprecise due to the small sample size, they have the advantage of avoiding bias due to unobserved heterogeneity.

## Conclusions

The effect of income and wealth on VPHI is non-linear. Only the top 40% of households show a greater proclivity to buy insurance, particularly the top quintile. Cross- sectional studies might bias the real effect upwards. Wealth is a relevant variable for explaining insurance decisions, but its effect is smaller than the effect of income.

## Supplementary information

**Additional file 1.** Semi-elasticity estimates using an imputed voluntary private health insurance price for each family. The table contains the estimation of income and wealth semi-elasticities of voluntary private health insurance.

## Data Availability

The datasets used from the Survey of Household Finances for this study are publicly available at: https://www.bde.es/webbde/en/estadis/infoest/temas/te_encuestas.html

## Abbreviations

NHS: National health service
OECD: Organization for economic co-operation and development
VPHI: Voluntary private health insurance
EEF: Survey of household finances (in Spanish)
cpf: Cumulative probability function
IMR: Inverse mills ratio
APE: Average of partial effects
COICOP: Classification of individual consumption according to purpose
OR: Odd-ratio
US: United States
