# Supplementary material for: Income and wealth as determinants of voluntary private health insurance: empirical evidence in Spain, 2008–2014
Source: BMC Public Health. 2020 Aug 19;20:1262. doi: 10.1186/s12889-020-09362-5 (PMC7437046; doi:10.1186/s12889-020-09362-5)
Supplement: Supplementary file 1 — Additional file 1. Semi-elasticity estimates using an imputed voluntary private health insurance price for each family. The table contains the estimation of income and wealth semi-elasticities of voluntary private health insurance. [file 12889_2020_9362_MOESM1_ESM.docx]

**Additional file 1.**

Cross-section and panel logit modes. Estimation of income and wealth semi-elasticities of voluntary private health insurance

| Wave | Nº of obs. | Income  (95% CI) | Wealth  (95% CI) | VPHI price (95% CI) |
| --- | --- | --- | --- | --- |
| 2008 | 6,197 | 0.066***  (0.026; 0.107) | 0.016***  (0.007; 0.025) | -0.035***  (-0.065; -0.005) |
| 2011 | 6,084 | 0.079***  (0.050; 0.108) | 0.008**  (0.002; 0.014) | -0.009  (-0.040; 0.022) |
| 2014 | 6,116 | 0.116***  (0.095; 0.138) | 0.006**  (0.001; 0.012) | -0.032**  (-0.059; -0.006) |
| Panel | 1,928 | 0.079*  (-0.013; 0.17) | 0.046  (-0.011; 0.102) | -0.234*  (-0.500; 0.323) |

The values in the table report the absolute change in the probability of having voluntary private health insurance if the income or wealth increases by 1%. All the models adjust only by education level of the head of the family and the expected future income. To avoid the presence of multicollinearity, the variables: age and sex of the head of the family, number of children under 14 years of age in the household, and proportion of people in the household with bad or very bad health were removed from the models. The income and wealth of the household are in logarithms and adjusted by family composition according to the OECD scale. For the conditional fixed effects logit model (panel data) the estimates are average (semi) elasticities of $P(Y_{iy}=1|X_{it},\alpha_{i})$, calculated following Kitazawa [22]. The last row contains the estimators of the panel model with household fixed effect.

*** significant at 1%; ** significant at 5%; * significant at 10%
